# Investigation of Outbreak-Specific Nonsynonymous Mutations on Ebolavirus GP in the Context of Known Immune Reactivity

**DOI:** 10.1155/2018/1846207

**Published:** 2018-11-15

**Authors:** Kerrie Vaughan, Xiaojun Xu, Bjoern Peters, Alessandro Sette

**Affiliations:** ^1^La Jolla Institute for Allergy and Immunology, 9420 Athena Circle, La Jolla, CA 92037, USA; ^2^University of California San Diego, Department of Medicine, La Jolla, CA 92093, USA

## Abstract

The global response to the most recent EBOV outbreak has led to increased generation and availability of data, which can be globally analyzed to increase our understanding of immune responses to EBOV. We analyzed the published antibody epitope data to identify regions immunogenic for humans on the main GP antigenic target and determine sequence variance/nonsynonymous mutations between historical isolates and variants from the 2013-2016 outbreak. Approximately half of the GP sequence has been reported as targeted by antibody responses. Our results show an enrichment of nonsynonymous mutations (NSMs) within epitopic regions on GP (70%, *p* = 0.0133). Mapping NSMs to human epitope reactivity may be useful for future therapeutic and prophylaxis development as well as for our general understanding of immunity against EBOV.

## 1. Introduction

During the 2013-2016 Ebola virus outbreak in West Africa, more than 28,000+ infections were reported, resulting in over 11,000 deaths [1, 2]. These figures from this outbreak far exceed the 286 total deaths from Ebola virus reported in the previous decade. The reasons for the unprecedented scale of the outbreak are likely multifactorial, including civil instability and challenges in diagnosis and response (medical infrastructure) [3–5].

Increased infectivity of the circulating virus strain (or variants thereof) has also been implicated. Molecular epidemiological studies of the EBOV outbreak variant, Makona, have revealed the emergence of genetically distinct viral lineages [6–11]. Sequence analysis of patient isolates (variants) revealed a number of nonsynonymous mutations (NSMs) in the Ebola virus (EBOV) genome [7, 12]. Subsequent studies have demonstrated that several substitutions were located within the sole surface glycoprotein (GP), which plays a critical role in infectivity and is a major target of humoral immune response. It is therefore possible that such mutations influenced transmission rates of and/or immunity to the EBOV variants circulating since 2013 [13, 14].

Characterizations reported to date of responses in EBOV patients following natural infection have established a relationship between early vigorous humoral and cellular immune responses and survival, including persistent neutralizing activity and IgG immunoreactivity, as well as an elevated cytokine response [15–17]. By contrast, fatal infections have been associated with poor IFN*γ* production, limited CD8^+^ T cell activation, and low levels of anti-EBOV IgG [18–22]. Studies of humoral responses in particular have consistently suggested a role for neutralizing antibodies in survival. Moreover, some of these antibodies have also been shown to possess a significant level of cross-reactivity [23, 24].

Since the 2013-2016 outbreak, only two EBOV vaccines have been licensed for use in humans, China's Ad5-EBOV and GamEvac-Combi developed in Russia [25, 26]. In addition, several candidate therapeutic and prophylactic measures have been utilized with varying success, including convalescent sera, monoclonal antibody cocktails (ZMAPP) [27, 28, 42], and antivirals (TKM-Ebola, Tekmira) [29]. Experimental vaccines tested to date in humans include ChAd3-ZEBOV [30] and rVSV-ZEBOV [31]. Numerous other candidate vaccines remain in the pipeline at various stages of development and/or preclinical testing [32]. Apart from TKM-Ebola, which targets viral proteins L, VP24, and VP35, all of these target GP, underscoring the importance of understanding the potential impact of changes at the molecular level for this critical antigen. Changes at the molecular level during major outbreaks can impact epitopic regions or sites that can then translate into functional adaptation favoring the virus.

The Immune Epitope Database (IEDB) is a repository for T cell and antibody epitopes reported from the published literature [http://www.iedb.org]. These data include epitopes defined in humans and animal models in the context of infectious disease, autoimmunity, and allergy. The IEDB therefore provides a unique resource for the analysis of EBOV immune reactivity at the molecular level. Herein, we analyze experimental antibody response data to investigate the potential implicates of NSMs on EBOV immunity pre- and postoutbreak.

## 2. Materials and Methods

### 2.1. GP Sequence Selection and Determination of Sequence Conservation for Ebolaviruses

**Figure 1:**
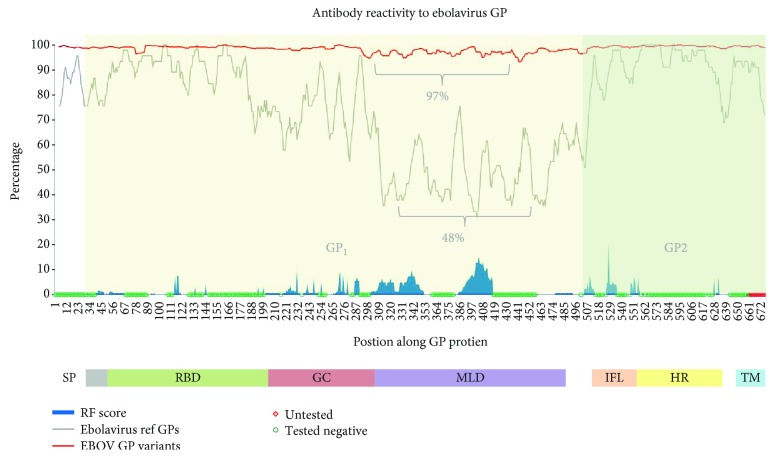
Mapping of antibody responses with sequence identity along the entire GP protein. Immunome Browser epitope mapping of all antibody responses per residue of the reference GP sequence (blue) compared with plot of sequence identity for ebolavirus GP NCBI reference sequences (grey) and all EBOV variants (red). Shaded regions represent the two subunits of the glycoprotein, GP_1_ aa33-501 (yellow) and GP_2_ aa502-676 (green). Region 1-33 represents the signal peptide (unshaded). Sequence identity is plotted as the running average with a window of 9. Sequence identities shown above represent the average for the region wherein responses are present (average of raw sequence ID scores). RFscores here were converted to percentages. Abbreviations: signal peptide: SP; receptor-binding domain (54-201): RBD; glycan cap (201-309): GC; mucin-like domain (305-485): MLD; internal fusion loop (524-539): IFL; heptad repeats (554-595, 615-634): HR; transmembrane region (651-671): TM.

The method for identifying and collecting ebolavirus sequence data was similar to the pipeline procedure developed and described in our previous work analyzing viruses in the *Flavivirus* genus [33]. The Entrez package from Biopython was utilized to query the NCBI protein data repository for full-length ebolavirus GP protein sequences (GP, sGP, or ssGP). These records were then processed to extract associated information (species, strain, and/or isolate/variant name, accession ID, year, and location).  Table S1 lists all sequences retrieved in this query, representing the set of full-length GPs available from NCBI. This set includes variants from the recent outbreak in West Africa, as well as sequences from previous outbreaks in different geographic locations. Following removal of sequences that occurred more than once, all unique GP sequences were aligned using MAFFT [34]. To analyze sequence conservation specifically among different *Ebolavirus* species, the following five reference sequences were used: Ebola virus (NP_066246.1), Sudan (YP_138523.1), Tai Forest (YP_003815426.1), Reston (infects NHP only; NP_690583.1), and Bundibugyo (YP_003815435.1); all complete genomic polyprotein (proteome) sequences with full annotation and associated metadata.  Figure 1 represents two separate multiple sequence alignments (MSAs) of GPs; one of the 5 representative ebolavirus species (in grey) and one of the EBOV variant sequences (in red) [193 in total; full-length, nonredundant].

### 2.2. IEDB Data Curation Methodology

Although the IEDB curation guidelines are detailed elsewhere [35], we reiterate some basics here that are relevant to the present analysis. Briefly, the IEDB uses automated document classifiers [36] to identify all articles indexed in PubMed that describe epitopes. For those scoring above a conservative threshold, the full text articles are retrieved and inspected by a curator who determines if original data specific to epitope recognition is included. One inclusion criterion is that the molecular structure of recognized epitopes was definitely identified at the molecular level (amino acid residue) and mapped to a region of 50 amino acids or smaller. For antibody responses, this includes linear stretches of amino acids, sets of discontinuous amino acids that form regions on a 3D protein structure, or even single residues, such as those defined by loss of function assays. As every journal article is curated separately, two epitopes are reported as distinct entities in the IEDB if they have any difference in molecular structures, even if they largely overlap. Thus, in many cases, the epitopes reported in different studies can overlap at the same antigenic site.

### 2.3. IEDB Data Retrieval

The IEDB web interface was used to query for all records (positive and negative outcomes) where the epitope source organism was within the *Ebolavirus* genus (NCBI taxonomic ID 186536) for B cell assays, thus excluding T cell assays and those associated with MHC ligands, and included the following fields associated with the records: epitope id, description (sequence), antigen name, position, antigen id (accession) epitope source organism name. For these general analyses, only epitopes identified in human hosts were considered. An exception was made for the “functional antibody” epitope dataset, for which we included epitopes identified in any host species for which the epitope recognizing antibodies were shown to have specific biological functions, namely, *in vitro* neutralization, antibody-dependent cellular cytotoxicity, complement-dependent cytotoxicity, and those representing *in vivo* challenge assays (protection from, survival from challenge, challenge decreased disease, and pathogen burden after challenge). This exception was made to take into account that such assays (especially *in vivo* assays) are essentially exclusively performed in animal model systems and to include those well-known monoclonal antibodies used recently in human treatment cocktails.

### 2.4. Alignment of Known Epitope Residue Data with GP Sequences and Calculation of RFscore

Epitope data from the genus *Ebolavirus* extracted from the IEDB (as described above) were first oriented for position to reference GP sequences; data to date come from only EBOV, Sudan virus, and Bundibugyo virus GPs and include isoforms GP and ssGP. Each epitope residue was first aligned to the reference genomic polyprotein sequences in order to identify the putative position of the epitope within GP (amino acid positions can vary among strains and variants). To ensure the accuracy of reported and mapped positions, the degree of sequence identity between each epitope and its mapped position on GP was calculated as the percentage of the identical residues in the epitope aligned region.

Once the epitope location on GP was established, a positional response frequency (RFscore) was calculated using previously established parameters (http://help.iedb.org/entries/91331263-Immunome-Browser-3-0). Response frequency data are provided by the authors (% of positive respondents) and are not generated by the IEDB. The Immunome Browser feature within the IEDB makes use of these data (when provided) to visually display responses to *tested* regions on an antigen. RF scores were determined from those assays in which the numbers of positive respondents to the epitope were reported out of the total number of subjects tested (e.g., 8 of 10 subjects responded). For a given residue in the protein sequence, data from all epitopes containing that residue were considered (at least 1 positive assay). To assign a higher weight to sequence regions that were extensively tested (larger N), and thus have a higher confidence in the calculated frequency of responding donors, the lower bound of the 95% confidence interval associated with that frequency was taken as the RFscore. In this way, the RFscore provided a measure of overall immunological prominence to certain residues or regions on an antigen.

### 2.5. Compilation of Nonsynonymous Mutations

The compilation of outbreak-specific NSMs on EBOV GP was accomplished through the identification of all sites reported previously in the literature [13, 37] [7, 9, 38, 39]. The analysis of NSMs with respect to antibody epitope sites was carried out statistically by establishing the relationship among positive sites with NSM, negative sites with NSM, positive sites with non-NSM, and negative sites with non-NSM.

### 2.6. Statistical Analysis

To determine the statistical relationship between NSM occurrence and epitope location, we employed two-tailed Fisher's exact test, which analyzes a two-by-two contingency table to calculate the association between groups and outcomes.

## 3. Results

### 3.1. Antibody Reactive Regions on GP Correspond to Regions of Higher Interspecies Sequence Variation

In order to gain a better understanding of immune reactivity with respect to GP sequence conservation and/or variation, we first mapped all published antibody epitopes (all hosts) as a function of their respective locations on GP. These data represented epitopes from EBOV, SUDV, and BUDV GPs (GP and ssGP). To do this, we used the Immunome Browser (IB) feature within the IEDB, which plots all response frequency (RFscores) data onto a reference antigen or proteome for each residue of the epitope. The IB feature thus visually displays all reactive (positive) and unreactive (tested negative) as well as untested regions on a given antigen. In this way, the IB provides a measure of immunodominance, as it reflects which residues or regions are recognized most frequently. Next, we overlaid the IB RFscore plot with the GP conservation data in order to visualize the overall epitope coverage with respect to all ebolavirus GPs and then to EBOV GP variants only.

Figure 1 shows antibody reactivity to GP proteins reported to date from ebolavirus species along the *x*-axis. Response data depicted here included human antibody responses (mostly polyclonal sera), as well as murine monoclonal antibodies, thus to our knowledge represent all sites defined to date for any host on GP. The graph reveals that the majority of GP has been evaluated empirically for antibody reactivity (as either positive or negative), as evident in the lack of any significant untested region. The graph shows two highly reactive regions in terms of breadth and response frequency: aa301-351 (50 residues with average RFscore 4.2) and aa385-417 (32 residues with average RFscore 7.7), with several narrower regions also showing high to moderate reactivity: aa526-535 (RFscore 6.1), aa286-290 (RFscore 5.9), aa268-276 (RFscore 3.6), aa549-557 (RFscore 3.7), 505-514 (RFscore 3.2), aa113-127 (RFscore 1.9), and aa201-251 (RFscore 1.3). Thus, while reactivity was observed in both GP_1_ and GP_2_ domains, responses in GP_1_ appear to be predominate. Several individual residues were also noted with high response score (range 3.1-7.1), including aa134, aa144, aa194, aa199, aa254, aa628, and aa632. These residues have been identified as being part of discontinuous monoclonal antibody epitopes.

Comparison of the antibody responses data to the alignment of reference ebolavirus GP sequences (grey line) revealed significant interspecies sequence variation (as low as 48% GP sequence identity) within the highly reactive sites on GP (aa301-351 and 385-417), whereas less sequence variation was observed for the other reactive sites, aa201-251, aa268-276, and aa286-290 (~70% GP sequence identity). By contrast, analysis of sequence variation within EBOV GP variants (red) shows a high degree of conservation, even within the highly reactive regions. Sequence variation within these reactive sites for EBOV GP is much lower (~3%). We conclude that the greatest degree of sequence variation appears to occur within regions on GP shown to be targets of antibody responses, and while interspecies (EBOV, Sudan, and Bundibugyo) variation at these sites is relatively high, variation *between* representative EBOV variants at these same sites is lower. This first analysis thus provided a “big picture” view of antibody reactivity with respect to GP sequence conservation and/or variation. It revealed that significant human antibody reactivity was focused in a region of GP with the greatest degree of variation among EBOV variants, including those representing viruses isolated during the 2013-2016 outbreak. While the overall degree of variation is low (~3%), this observation is intriguing and provides rationale for examining a potential role for immune pressure in GP sequence variation.

### 3.2. Nonsynonymous Mutations Identified in Makona-Lineage EBOV Are Disproportionately Found within the Antibody Reactive Regions on GP

To further examine a potential role for immune pressure in GP sequence variation, we sought to identify all possible nonsynonymous mutations (NSMs) to have occurred within the 2013-1016 outbreak (EBOV Mayinga versus Makona lineages). Previous studies investigated NSMs and the potential for functional changes that might lead to increased viral fitness [7, 12, 13, 40]. These studies established that a relatively high level of sequence variation is present, when comparing among the 2013-2016 EBOV outbreak variants, which is consistent with functional adaptation in humans [40].

To investigate whether some viral adaptations may lead to immune escape/evasion, we addressed whether NSMs reside within regions previously tested for antibody reactivity in humans. A survey of the literature uncovered a total of 59 NSMs identified to date within GP from EBOV Makona phylogeny, listed in Table 1 [7, 9, 13, 14, 37–39, 41]. Using the ebolavirus GP-related antibody data captured in the IEDB as of April 2018, we wanted to determine whether these NSMs fell within regions defined previously as immunoreactive in human subjects.

In all, 353 residues were derived from immunogenic regions and 306 from nonimmunogenic regions (just 17 residues untested). We found that 70% (41/59) of the identified NSMs fall within epitope regions (site resides within positive linear or discontinuous epitope), whereas 30% (18/59) are located within nonimmunogenic regions (tested and found to be negative in all instances) (Table 1). The significance of these differences was established by an exact Fisher test which was associated with a two-tailed *p* value of *p* = 0.0133. Moreover, of the 41 sites found to be positive and containing NSMs, 68% of these [24] fall within the highly immunogenic regions shown in Figure 1 (regions with the highest RFscores). Nearly all of these sites are located on the GP_1_ subunit (33-501) and within the mucin-like domain (MLD; 305-485). All data represent sites identified using human sera, with the exception of T411A, G286R, H389R, L479P, T485A, and I486T, which are part of the epitopes recognized by the well-known murine monoclonal antibodies, 13F6, considered for use as part of a therapeutic mAb cocktail MB-003 [42, 43], FVM09 [44], and 6D8, 14G7/12B5 1-1 [45], respectively. Interestingly, NSM at residue 82 (A82), which in some studies has been shown to be associated with increased mortality and thought to heighten intrinsic infectivity of the virus [13, 37], was found to be a nonepitope (tested negative). One site (N107D) fell within the receptor-binding domain, and another site (T272A) lies within glycan cap (268-278, 299-310), which is a known target of neutralizing antibodies [23]. In all, 18 of these NSM sites are located in regions associated with *in vitro* virus neutralization and/or *in vivo* protection (demonstrated in animal models of infection).

This analysis thus revealed that the vast majority of identified NSMs arising following the 2013-2016 outbreak fall within the reported epitopic regions. Nearly all of the sites showing the greatest response frequency score were located on the GP_1_ subunit and within the MLD (305-485). These findings therefore suggest a potential role of antibody responses in influencing EBOV GP sequence variation.

## 4. Conclusions

In this work, our aim was to investigate two related aspects of ebolavirus GP immunobiology, firstly presenting a “big picture” view of all antibody reactivity against ebolavirus GP in the context of sequence variation and secondly to evaluate this reactivity with respect to nonsynonymous mutations identified following the West African outbreak. The goal was to gain a better understanding of the nature of human reactivity and potential sites of immune pressure. Using data collected from a series of studies identifying NSMs generated during the 2013-2016 outbreak in West Africa that are distinct from historic ebolavirus EBOV variants (preoutbreak) [7, 12, 13, 38, 40] and antibody epitope data cataloged within the IEDB, we found that significant human antibody reactivity was focused in a region of GP with the greatest degree of variation and that these sites overlapped significantly with NSMs. Of note, a similar analysis of T cell responses could not as yet be performed due to the lack of sufficient published data. This lack of EBOV-specific human T cell epitope data represents a significant knowledge gap warranting future investigation.

Until now, reports on NSMs have mostly focused on functional changes that would affect virus fitness/transmission, with the exception of Park et al., wherein NSMs were mapped to therapeutic and diagnostic sites [39]. In this report, we present an analysis of EBOV sequence variation between pre- and postoutbreak variants as it pertains to antibody reactivity at the molecular level for human subjects, including as well sites from known therapeutic monoclonal antibodies, and identify the location of all GP NSMs reported to date with respect to positive and negative data.

We first established the degree of sequence variance for GP with respect to published antibody response data, utilizing the IEDB's Immunome Browser feature to map positive and negative response data. We then superimposed these response data against GP sequence variance. Using multiple sequence alignment of available species, we found that the greatest degree of sequence variance occurs within regions on GP shown to be targets of antibody responses (regions of high response frequency), and while interspecies variance at these sites is relatively high (e.g., EBOV, Sudan, Bundibugyo, and Tai Forest), variance *between* representative EBOV variants (those involved in recent outbreak) at these same sites is comparably lower. This is similar to what is previously reported for the monoclonal antibodies cocktails ZMAPP, MB-003, and ZMAB [46]. Nevertheless, our novel observation was that several of the immunodominant regions (defined as those regions with high response frequency) correspond to regions of the EBOV GPs that shows increased variance (aa291-510).

We next sought to determine the extent to which NSMs were located within antibody reactive/immunodominant sites. We therefore mapped all NSMs reported to date between the “Mayinga” (preoutbreak) and “Makona” (postoutbreak) lineages and evaluate the extent to which these residues fell within known epitopic sites on GP (within any region tested positive). We found that 70% (41/59) of the identified NSMs fell within epitope regions (site resides within positive linear or discontinuous epitope), whereas 30% (18/59) were located within nonimmunogenic regions (tested and found to be negative in all instances). Nearly all of these sites were located on the GP_1_ subunit (33-501) and within the MLD (305-485). Thus, our study may suggest a potential role of antibody responses in influencing EBOV GP sequence variation.

In evaluating the possible functional significance of these data, we focused on the nature of the human antibody sites on GP, which shows that the majority of overlapping NSM/epitope sites are located within the MLD. The MLD comprises aa305-485 of GP and contains numerous glycosylation sites (N- and O-linked). The MLD has been identified as the central disordered section on the GP protein [47]. Disordered regions have been implicated in providing an avenue through which viruses can take advantage of host perturbations through “sticky” interactions with host proteins [40, 48–50]. Therefore, it is conceivable that mutations in this “reactive” region are part of this mechanism. While this observation provides rationale for our current discussion, there are several salient points to address related to the GP/MLD in EBOV pathogenesis and immunobiology.

Until only recently, the MLD has been cited as the major target of EBOV-specific antibody response [45, 51, 52]. However, additional human antibody sites within the core/stalk, GP_1_, and GP_2_ as well as the glycan cap have also been described following infection [53, 54], suggesting a more complex picture of immunoreactivity against EBOV GP and underscoring exactly which regions on GP are truly immunodominant in humans that are yet to be fully characterized. It is important to note that the dominance of the MLD domain reported in earlier historical studies may actually be related to the use of improperly folded GP as an immunogen for epitope mapping. Indeed, recent studies of human antibodies from human survivors did not find dominance for the MLD, though this domain remains one of the three major targets of human antibody reactivity along with the glycan cap and GP_1_/core [53].

Since the completion of this analysis, two relevant papers have been published analyzing human antibody responses to infection (IIinkh 2018; Flyak 2018). In IIinkh et al., human monoclonals previously defined following natural infection with Bundibugyo virus ([23]; included in this study), all of which target the glycan cap and GP_2_ stalk region, were characterized with respect to their mechanisms of antiviral effects. Similarly, in Flyak et al., six human monoclonals are shown to specifically target the HR2-MPER region of GP_2_. Of note, all of these sites were considered in the present analysis. None of these stalk- or glycan cap-specific residues represent NSM sites identified thus far. It is important to reiterate here that these data represent all human antibody epitope sites reported to date in the published literature; most of which are polyclonal in nature and mostly overlap with known murine monoclonal antibody residues.

From the standpoint of *in vivo* pathogenesis, there is evidence suggesting that MLD could be among the important immune targets in human infection. During viral entry, the MLD and glycan cap facilitate viral adhesion to host cells, leading to macropinocytosis [55, 56]. Only after incorporation into the endosome are these moieties removed by host protease cleavage [57, 58]. Following budding from the host cell, mature virions contain intact GP, including the MLD and glycan cap. Further, GP has been shown to exert direct cytopathic effects on host cells, and MLD is required for this effect. MLD has also been shown to play a role in inflammatory dysregulation, immune suppression, and vascular damage leading to viral spread [59–65]. By contrast, studies in tissue culture have shown that deletion of the MLD from GP does not prevent entry of the virus into cell lines, suggesting that this region is dispensable [66, 67]. It is therefore possible that nonsynonymous mutations occurring in the MLD may predominate because this region is nonessential for infectivity. However, whether or not this domain is unessential in the course of natural human infection has yet to be shown. Interestingly, residues shown previously to be important for viral entry into the host cell, L57, L63, R64, G87, F88, K95, K114, K115, K140, G143, P146, C147, F153, H154, F159, F160, Y162, and I170 [68–70], were not found to overlap with the NSM sites analyzed herein.

Additionally, antibodies directed at the MLD have been shown to be nonneutralizing, presumably due to the cleavage of this domain from GP during the process of endocytosis, prior to receptor binding. However, it has been hypothesized that nonneutralizing antibodies targeting the MLD still play a role in reducing viral infectivity by binding GP at the surface and facilitating other protective responses, such as those mediated by antibody-dependent cytotoxicity (ADCC) or complement [71]. Indeed, many of the MLD-specific antibodies have been shown to be protective [45], providing evidence that these antibodies are preventing or quelling infection through means other than neutralization. Indeed, recent exhaustive characterization of 168 monoclonal antibodies as part of the Viral Hemorrhagic Fever Immunotherapy Consortium [VIC; http://vhfimmunotherapy.org] efforts reveals that protective responses are mediated through both Fab (neutralizing) and Fc-driven processes (nonneutralizing) [53, 54, 72]. This analysis included neutralizing and nonneutralizing murine and human monoclonals generated prior to the outbreak, as well as a smaller number donated after the event. While the epitope residues associated with this panel were not reported therein, they represent a wealth of information of reactivity at the molecular level, thus making future collaboration with VIC highly desirable.

The analysis presented herein, whereby the cumulative epitope data were investigated for their relationship to all nonsynonymous mutations identified from the 2013-2016 EBOV outbreak in West Africa, suggests a possible association between sites of human antibody reactivity against GP and these sites of variation. The cumulative data also suggest that the MLD may be a specific target of immune activity within the context of NSM. Evidence to date cannot rule-out a significant role for the MLD in infectivity and immunomodulation during EBOV pathogenesis in humans, helping to promote EBOV infection, thus making the occurrence of multiple NSM within regions of known immune reactivity within the MLD intriguing. While the exact implications of these findings are as yet unclear, we feel that these data highlight an association that warrants further investigation. Indeed, a more complete understanding of immunodominant regions on EBOV GP will only come from additional epitope mapping of human antibody reactivity following natural infection.

Finally, it is important here to reemphasize the original motivation for this investigation and therefore to hopefully place these findings in the broader context. The 2013-2016 West African EBOV outbreak was entirely unprecedented in its scope and overall human toll. Indeed, the total number of people affected (morbidity) far exceeded numbers tallied for all previous outbreak *combined* going all the way back to the first documented outbreak in 1976. While the forces behind this event are likely multifactorial, the overall breadth of the outbreak suggests that factors related to the virus *itself* were at play. Whether these changes are related to immune pressure, representing changes within immunodominant sites still remains to be elucidated.

## Figures and Tables

**Table 1:** Correspondence of epitope data with nonsynonymous mutations on EBOV GP.

NSM^∗^	Response	IEDB antibody data						Mutational analyses	
		Epitope ID (IEDB)	Host	Positive	Negative	GP location	Functional	Previous^∗^	Outbreak^∗^
29	Nonepitope	40693, 187386	H	NA	**R**	SS	NA	**R**	**K**
31	Nonepitope	227630, 40693, 187386, 227534	H	NA	**F**	SS	NA	**F**	**S**
46	**Epitope**	61752	H, M (16F6)	**S**	NA	GP1	**N**	**S**	**N**
47	**Epitope**	61752, 1902944	H, M (16F6)	**E**	NA	GP1	**Y**	**E**	**D**
82	Nonepitope	57019, 187449, 227411	H	NA	**A**	GP1; RB	NA	**A**	**V**
107	**Epitope**	227450^∗∗∗^	H	**N**	NA	GP1; RB	N	**N**	**D**
202	**Epitope**	13781	H	**P**	NA	GP1	N	**P**	**L**
206	**Epitope**	13781	H	**T**	NA	GP1	N	**T**	**M**
212	**Epitope**	13781	H	**G**	NA	GP1	N	**G**	**D**
213	**Epitope**	13781	H	**Y**	NA	GP1	N	**Y**	**H**
214	**Epitope**	13781	H	**Y**	NA	GP1	N	**Y**	**H**
222	**Epitope**	187549, 50368	H	**A**	NA	GP1	N	**A**	**V**
230	**Epitope**	50368, 187549, 227170	H	**T**	NA	GP1	N	**T**	**A**
239	**Epitope**	227097	H	**L**	NA	GP1	N	**L**	**S**
262	**Epitope**	502927	H	**T**	NA	GP1	**Y**	**T**	**A**
272^^^	**Epitope**	442032	M (MB-003/13C6)	**K**	NA	GP1	**Y**	**K**	**N**
283^^^	Nonepitope	187578, 25693	N	NA	**T**	GP1	NA	**T**	**A**
286	**Epitope**	478550	NHP (mAb FVM09)	**G**	NA	GP1	**Y**	**G**	**R**
291	Nonepitope	187262, 25693	H	NA	**W**	GP1	NA	**W**	**R**
314	Epitope′	28398, 227137, 227646, 227406	H	**G**	NA	GP1; M	N	**E**	**K/D**
315	**Epitope**	28398, 227137, 227646, 227406	H	**A**	NA	GP1; M	N	**A**	**P**
326	Epitope′	227409, 52394, 187509, 227406	H	**T**	NA	GP1; M	N	**L**	**P**
330	**Epitope**	227409, 52394, 187509, 227099	H	**P**	NA	GP1; M	N	**P**	**S**
331	**Epitope**	227409, 52394, 187509, 227099	H	**G**	NA	GP1; M	N	**G**	**R**
336	**Epitope**	187509, 227099, 227422	H	**T**	NA	GP1; M	N	**T/L**	**N/M**
354	Epitope′	27448, 227141, 187269, 227664	H	**H**	NA	GP1; M	N	**T**	**I**
359	Epitope′	227141, 187269, 227664, 227225	H	**E**	NA	GP1; M	N	**G**	**K**
367	Nonepitope	227664, 227225, 5540, 187276, 227378, 64732	H; M	NA	**T**	GP1; M	NA	**T**	**A**
371	Nonepitope	64732; 227052	H; M	NA	**I**	GP1; M	NA	**I**	**V**
375	Nonepitope	64732, 227052, 66371, 227635	H; M	NA	**P**	GP1; M	NA	**P**	**S**
382	**Epitope**	227307	H	**P**	NA	GP1; M	N	**P**	**T**
389	**Epitope**	187300, 233197, 227248	H; M (6D8)	**H**	NA	GP1; M	**Y**	**H**	**R**
395^^^	**Epitope**	227307, 187300, 233197, 227248, 65808, 227687, 442034	H; M (6D8)	**K**	NA	GP1; M	**Y**	**K**	**R/G/E**
397^^^	**Epitope**	227307, 187300, 233197, 227248, 65808, 227687, 442034, 8777, 187296	H; M (6D8)	**D**	NA	GP1; M	**Y**	**D**	**G**
398	**Epitope**	227687, 442034, 187296	H; M (6D8)	**I**	NA	GP1; M	**Y**	**I**	**T**
405	**Epitope**	8777; 187296; 233152; 68320; 13837	H; M (13F6)	**E** ^∗∗^	NA	GP1; M	**Y**	**E**	**G**
406^^^	**Epitope**	13837, 442031, 68320, 65808, 227687, 8777, 187296, 233152	H; M (13F6)	**Q**	NA	GP1; M	**Y**	**Q**	**R**
407	**Epitope**	13837; 68320; 8777; 187296; 233152	M (13F6)	**H**	NA	GP1; M	**Y**	**H**	**Y**
410	**Epitope**	13837; 68320; 8777; 187296; 233152	M (13F6)	**R**	NA	GP1; M	**Y**	**R**	**S**
411	**Epitope**	442031	M (13F6)	**T**	NA	GP1; M	N	**T**	**A**
419	Nonepitope	52688, 227526, 227403, 227048	H	NA	**D**	GP1; M	NA	**D**	**E**
430	Nonepitope	227048, 49300, 227596, 227449	H	NA	**P**	GP1; M	NA	**P**	**L**
439	Nonepitope	227596; 1641; 227449; 227142; 227633	H	NA	**K**	GP1; M	NA	**K**	**E**
440	Nonepitope	1641, 227449, 227142, 227633	H	NA	**S**	GP1; M	NA	**G**	**S**
441	Nonepitope	227449, 227142, 227633, 63152	H	NA	**T**	GP1; M	NA	**T**	**A**
443	Nonepitope	227449, 227142, 227633, 63152	H	NA	**F**	GP1; M	NA	**L**	**S**
446	Nonepitope	227142; 227633; 63152; 187301	H	NA	**P**	GP1; M	NA	**P**	**L**
448	Nonepitope	227633, 63152, 187301	H	NA	**T**	GP1; M	NA	**T**	**A**
455	Nonepitope	63152, 187301, 227493, 227641	H	NA	**H**	GP1; M	NA	**H**	**Y**
462	**Epitope**	156605	M (ZMAPP/4G7)	**N**	NA	GP1; M	**Y**	**N**	**Y**
472	**Epitope**	187541	H	**E**	NA	GP1; M	N	**E**	**G**
479	**Epitope**	187541, 162327	H; M (12B5 1-1)	**L**	NA	GP1; M	**Y**	**L**	**P**
480	**Epitope**	162327; 187541	H; M (14G7)	**G**	NA	GP1; M	**Y**	**G**	**D**
485	**Epitope**	162327	M (14G7; 12B5 1-1)	**T**	NA	GP1; M	**Y**	**T**	**A**
486	**Epitope**	162327	M (14G7; 12B5 1-1)	**I**	NA	GP1	**Y**	**I**	**T**
499	**Epitope**	156605	M (ZMAPP/4G7)	**T**	**T**	GP1	**Y**	**A**	**T**
503	**Epitope**	549302	M (4G7)	**A**	NA	GP2	N	**A**	**V**
545	**Epitope**	549302	H	**E**	NA	GP2	N	**E**	**D**
637	Nonepitope	51081; 187484; 227088; 68004; 227315; 187302	H; M	NA	**D**	GP2	NA	**D**	**G**

*NSM*: residue site of nonsynonymous mutation; *Response*: results from Immunome Browser mapping performed April 25, 2017; *Epitope ID* refers to individual, unique epitopes reported within the IEDB. More than one ID = more than one epitope (positive or negative); ^∗^NSM and residue data summarized from [7, 9, 39, 38, 13, 14, 37]. All data are from GP or ssGP; *Location*: region on GP; *Functional*: designation for epitopes defined in the context of functional assay (virus neutralization/inhibition shown *in vitro* and/or protective *in vivo*). Nonepitope = tested and found to be negative (nonimmunogenic versus untested). ^∗∗^Previously analyzed in [46]. RB: receptor-binding domain (54-201); M: mucin-like region (305-485). GP1 (33-501); GP2 (502-676); M: mouse Ab; H: human sera; ^^^NSMs identified by [41] following failed MB-003 mAb cocktail trial in NHP.

## Data Availability

The human antibody data used to support the findings of this study (Figure 1 and Table 1) are included within the article. The resource from which these data were derived and analyzed, the Immune Epitope Database (IEDB), is accessible at http://www.iedb.org. All ebolavirus sequence data (listed in Table S1) were obtained through NCBI Protein database query (including criteria: txid186536Organism AND (glycoproteinTitle or GPTitle or sGPTitle or ssGPTitle) NOT srcdb pdbProperties).

## Supplementary Materials

Supplementary Materials Table S1: list of all selected strain/variant names, accession IDs, GI numbers, percent match to consensus sequence and country, host, and date of isolation, if known.
